# Epidemiology of Lyme Disease Diagnoses among Older Adults, United States, 2016–2019^1^

**DOI:** 10.3201/eid3009.240454

**Published:** 2024-09

**Authors:** Amy M. Schwartz, Christina A. Nelson, Alison F. Hinckley

**Affiliations:** Centers for Disease Control and Prevention, Fort Collins, Colorado, USA (A.M. Schwartz, C.A. Nelson, A.F. Hinckley);; University of Iowa, Iowa City, Iowa, USA (A.M. Schwartz)

**Keywords:** Lyme disease, Medicare, incidence, elderly, United States, tickborne diseases, vector-borne infections, bacteria

## Abstract

We used Medicare data to identify >88,000 adults >65 years of age diagnosed and treated for Lyme disease during 2016–2019 in the United States. Most diagnoses occurred among residents of high-incidence states, in summer, and among men. Incidence of diagnoses was substantially higher than that reported through public health surveillance.

Lyme disease (LD) is the most reported vector-borne disease in the United States (*1*). In separate efforts designed to better measure the burden of disease in the United States, we used employer-sponsored insurance claims data to quantify LD diagnoses (*2*,*3*). However, those data lacked information on persons ≥65 years of age, a group commonly affected by LD (*2*,*3*). In this complementary effort we used similar methods to analyze Medicare fee-for-service (FFS) data to describe LD diagnoses among beneficiaries ≥65 years of age.

## The Study

We examined Medicare FFS claims data and Part D drug event data (Appendix) to identify LD diagnoses. We restricted the analyzed population to Medicare FFS beneficiaries ≥65 years of age who participated in Parts A, B, and D for at least 12 months of a calendar year or until their month of death during 2016–2019.

Aligning with previously described methods (*2*), we defined LD diagnosis as an International Classification of Diseases, 10th Revision, Clinical Modification code for LD combined with a drug claim for an antibiotic indicated for LD (Appendix) within 30 days of the LD code and prescribed for >7 days. We defined an inpatient LD diagnosis as hospitalization with a primary code for LD or a primary code for a known manifestation or plausible co-infection of LD plus a secondary code (A69.2x) for LD (Appendix) (*2*,*3*).

We compared the restricted Medicare FFS study population with 2019 US Census estimation data for persons >65 years of age to ensure the 2 groups were similar with respect to sex, age, race, ethnicity, and region (*4*). We grouped states into incidence regions based on previous definitions (*3*).

We compared LD diagnoses identified in the Medicare FFS data to confirmed and probable cases among persons >65 years of age reported through national LD surveillance (*5*). State and local health departments investigate and tabulate LD surveillance cases and classify them according to the Council of State and Territorial Epidemiologists (https://www.cste.org) case definition in effect during the reporting year (*5*). We used SAS 9.4 and SAS Enterprise Guide 7.1 (SAS Institute Inc, https://www.sas.com/en_us) for analyses. The Centers for Disease Control and Prevention deemed this activity not research.

### Census Population and Medicare FFS Restricted Population

After restricting by enrollment criteria, we found that the Medicare FFS population had a median 17,872,466 person-years and the Census population had a median 51,561,372 persons during the study period (Appendix Figure 1). Compared with the Census population, the Medicare FFS population was slightly older (median age 74 years vs. 73 years), included more women (median 59.3% vs. 55.6%) (Appendix Figure 2), and featured a larger percentage of White/non-Hispanic persons (83.8% vs. 76.8%). The Medicare FFS population had a larger percentage of beneficiaries from states neighboring high-incidence states (median 28.0%) compared with the Census population (26.5%) and a lower percentage of beneficiaries from low-incidence states (47.5% vs. 51.4%). The characteristics of the Medicare FFS population remained stable during the study period.

### Characteristics of LD Diagnoses

We identified 88,485 LD diagnoses among Medicare FFS beneficiaries during 2016–2019, noting an average incidence of 123.5 diagnoses/100,000 person-years. We calculated a total of 34,183 LD cases reported through surveillance during 2016–2019, and an average incidence of 16.6 cases/100,000 persons (Appendix Figure 3).

### Geographic Distribution

Approximately 82% of LD diagnoses were among residents of high-incidence states (Table). The median incidence of LD diagnoses was 346.9/100,000 person-years among residents of high-incidence states, 35.3/100,000 person-years among residents of states or jurisdictions neighboring high-incidence states, and 29.4/100,000 person-years among residents of low-incidence states. In comparison, 93% of LD surveillance cases were among residents of high-incidence states. The median incidence of those cases was 57.1/100,000 persons among residents of high-incidence states, 3.6/100,000 persons among residents of states or jurisdictions neighboring high-incidence states, and 0.6/100,000 persons among residents of low-incidence states.

**Table:** Characteristics of Lyme disease diagnoses according to Medicare fee-for-service claims data versus cases identified by national surveillance in a population of persons > 65 years of age, United States, 2016–2019*

Characteristic	High-incidence states			Neighboring states			Low-incidence states	
	Medicare	Surveillance		Medicare	Surveillance		Medicare	Surveillance
Person-years	72,298	NA		5,958	NA		10,009	NA
Diagnoses or cases, no.	72,455	31,879		5,978	1,714		10,052	590
Diagnoses or cases, %	81.9	93.3		6.7	5.0		11.3	1.7
Incidence among men	422.9	71.0		38.3	4.6		30.1	0.6
Incidence among women	321.3	46.6		33.4	3.0		29.2	0.5
Occurring in May–August, %	59.5	72.2		58.2	79.9		45.8	66.2
Median incidence, 2016–2019 (range)	346.9 (337.2–417.8)	57.1 (52.9–65.6)		35.3 (27.9–36.6)	3.6 (2.4–5.3)		29.4 (27.7–31.6)	0.6 (0.5–0.6)

*Incidence calculated as diagnoses/100,000 person-years in Medicare fee-for-service or cases/100,000 population among each surveillance subcategory. NA, not applicable.

### Seasonality

**Figure 1:**
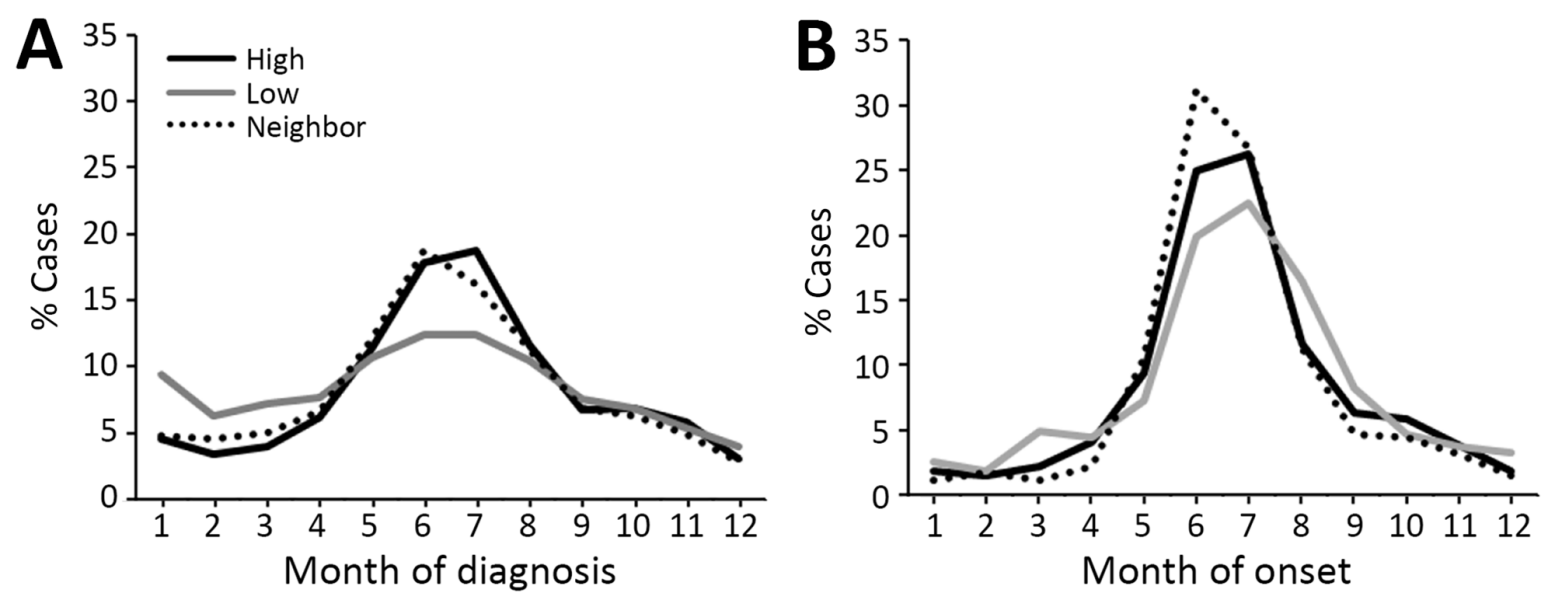
Analysis of Lyme disease among older adults, United States, 2016–2019. A) Percentage of Lyme disease diagnoses by month according to Medicare fee-for-service data. B) Percentage of Lyme disease cases by month of onset from US surveillance data.

Most (57.8%) LD diagnoses occurred during May–August, but most (72.6%) LD surveillance cases had onset during the summer months (Table; Appendix Figure 4). Compared with Medicare data, the peak in surveillance cases was more prominent for all regions. In addition, a larger proportion of LD diagnoses occurred in winter months among residents of low-incidence areas (Figure 1).

### Sex Distribution

Most (56.1%) LD diagnoses occurred among men; slightly more (60.4%) men were represented in surveillance cases. The median annual incidence of LD diagnoses among male Medicare beneficiaries was 134.3 diagnoses/100,000 person-years (range 131.6–160.5 diagnoses/100,000 person-years); median annual incidence of LD diagnoses among female beneficiaries was 109.5 diagnoses/100,000 person-years (range 103.3–125.7 diagnoses/100,000 person-years). In comparison, according to surveillance data, median annual incidence among men was 19.6 cases/100,000 persons (range 17.8–22.5/100,000 persons), and median annual incidence among women was 13.2 cases/100,000 persons (range 12.3–14.9/100,000 persons).

### Age and Sex by Region

**Figure 2:**
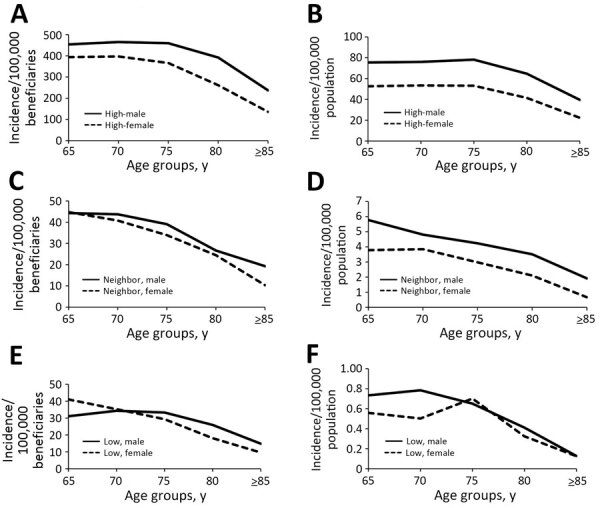
Lyme disease incidence for older adults, United States, 2016–2019. Results according to age group, sex, and geographic category of Lyme disease endemicity based on Medicare fee-for-service beneficiary data (A, C, F) and from US surveillance data (B, D, F). A, B) Disease incidence for men and women in high-incidence states. C, D) Disease incidence for men and women in neighboring states. E, F) Disease incidence for men and women in low-incidence states. Incidence calculated as diagnoses/100,000 beneficiaries in Medicare fee-for-service plans or cases/100,000 population among each subcategory. Scales for each y-axis differ substantially to underscore overall age-related incidence patterns but do not permit direct comparison of the magnitude of Lyme disease between systems or geographic categories.

In high-incidence states, men had the highest incidence of LD for all age groups in both Medicare and surveillance data (Figure 2). In neighboring states, according to Medicare data, women had a slightly higher incidence than men in only 1 age group (65–69 years), whereas according to surveillance data men had a higher incidence of LD across all age groups. In low-incidence states, according to Medicare data, women had a slightly higher incidence than men in 1 age group (65–69 years); in surveillance data, women had a higher incidence than men in a single age group (75–79 years).

## Conclusions

Among persons >65 years of age, epidemiologic trends for sex, age, and region are similar for Medicare diagnoses and cases identified through public health surveillance. Nevertheless, overall diagnoses per person-year are ≈7-fold higher than for LD incidence in surveillance data. Those findings align with findings reported in our previous claims analysis (*3*). Seasonality of LD differed somewhat by region when comparing Medicare FFS and surveillance data: high-incidence states and neighboring states exhibited similar patterns for diagnoses and surveillance cases, but low-incidence states demonstrated a more muted peak for LD diagnoses in summer. Those findings also aligned with past claims analyses (*2*,*3*).

Some differences exist between this study and previous claims analyses (*2*,*3*). Within the Medicare population, men had a higher incidence of LD compared with women in all age groups in the high-incidence and neighboring states. In past claims analyses, male incidence was higher in children (both regions) and older adults (high-incidence states). Overdiagnosis of LD has been previously reported (*6*–*9*) and may contribute to some of those differences.

We used methods similar to past claims analyses to identify LD diagnoses (*2*,*3*); those stated limitations also apply here. However, the source from which our present data are derived is different from past analyses, and its representativeness is a strength. Nearly all adults >65 years of age enroll in either the Medicare FFS program or the Medicare Advantage program, making Medicare FFS a reliable and consistent, though still incomplete, source of data for most US citizens in this age group. We noted some differences between the Medicare FFS population and the Census population regarding race, ethnicity, and sex, but differences were small and did not fluctuate over the study period.

In conclusion, we found that LD diagnoses identified from the Medicare FFS databases exhibit similar patterns to those of surveillance data, and that most diagnoses occur among residents of high-incidence states, in summer months, and among male beneficiaries. These findings, combined with data gathered in similar analyses, add insight into LD patterns that are unique to this older population in the United States. 

Appendix More information for epidemiology of Lyme disease diagnoses among older adults, United States, 2016–2019.
