# Supplementary material for: Epidemiology of Lyme Disease Diagnoses among Older Adults, United States, 2016–2019
Source: Emerg Infect Dis. 2024 Sep;30(9):1926–9. doi: 10.3201/eid3009.240454 (PMC11346992; doi:10.3201/eid3009.240454)
Supplement: Appendix — More information for epidemiology of Lyme disease diagnoses among older adults, United States, 2016–2019. [file 24-0454-Techapp-s1.pdf]

Article DOI: http://doi.org/10.3201/eid3009.240454

EID cannot ensure accessibility for supplementary materials supplied by authors. Readers who have difficulty accessing supplementary content should contact the authors for assistance.

Epidemiology of Lyme Disease Diagnoses among Older Adults, United States, 2016–2019

Appendix

Sources of Data

We examined inpatient base claim files, hospital outpatient base claims files, and carrier claims files which included International Classification of Disease, Revision 10, Clinical Modification Codes (ICD-10-CM) to identify Lyme disease-specific diagnosis codes and Part D drug event files to identify relevant treatment information (Appendix Table). The Master Beneficiary Summary File contained monthly plan enrollment information on all Medicare beneficiaries.

Supplemental Information

Drugs considered to be Lyme disease treatment as part of inclusion criteria for outpatient Lyme disease diagnoses:

- Amoxicillin
- Amoxicillin/clavulanic acid*
- Azithromycin or azithromycin dihydrate (intravenous forms excluded)
- Doxycycline (all forms)
- Cefotaxime sodium
- Ceftriaxone sodium

- Cefuroxime axetil
- Cefuroxime sodium*
- Clarithromycin
- Erythromycin—all forms except lactobionate (IV), gluceptate (IV), thiocyanate (not available in the United States), and ethylsuccinate/sulfisoxazole. Topical forms were also excluded.
- Minocycline hydrochloride (intravenous forms excluded)*
- Penicillin G (benzathine, procaine, or potassium)
- Tetracycline hydrochloride*

*These antimicrobial drugs are not formally recommended for treatment of Lyme disease but are closely related to the recommended drug or are a known historical treatment that some practitioners might still prescribe.

Appendix Table. ICD-10-CM codes used to identify Lyme disease diagnoses in the Medicare fee-for-service databases

Manifestation	ICD-10 Codes
Lyme disease	A69.20, A69.21, A69.22, A69.23, A69.29
Facial palsy	G51.0, G51.8, G51.9, G52.7, G52.8, G52.9, G53, R29.810, S04.50XA, S04.51XA, S04.52XA, S04.891A, S04.892A, S04.899A, S04.9XXA
Lyme carditis	I30.1, I30.9, I30.0, I30.8, I41, I40.9, I40.0, I40.1, I40.8, I51.4, I49.5,, I44.2, I44.30, I44.0, I44.1, I44.4, I44.5, I44.60, I44.69, I44.7, I45.0, I45.10, I45.19, I44.30, I44.39, I45.4, I45.2, I45.3, I45.5, I45.89, I45.9
Meningitis	G03.9, G01, G00.9, G00.8, G04.2, G03.0
Arthritis	M00.80, M00.9, M00.811, M00.812, M00.819, M00.821, M00.822, M00.829, M00.831, M00.832, M00.839, M00.841, M00.842, M00.849, M00.851, M00.852, M00.859, M00.861, M00.862, M00.869, M00.871, M00.872, M00.879, M00.88, M00.89, M01.X0, M02.80, M01.X11, M01.X12, M01.X19, M02.811, M02.812, M02.819, M01.X21, M01.X22, M01.X29, M02.821, M02.822, M02.829, M01.X31, M01.X32, M01.X39, M02.831, M02.832, M02.839, M01.X41, M01.X42, M01.X49, M02.841, M02.842, M02.849, M01.X51, M01.X52, M01.X59, M02.851, M02.852, M02.859, M01.X61, M01.X62, M01.X69, M02.861, M02.862, M02.869, M01.X71, M01.X72, M01.X79, M02.871, M02.872, M02.879, M01.X8, M02.88, M01.X9, M02.89, M01.X21, M01.X22, M01.X29, M02.822, M01.X32, M01.X39, M01.X51, M01.X52, M02.851, M01.X61, M01.X69, M02.861, M02.869, M01.X71, M01.X29, M01.X39, M01.X49, M01.X59, M01.X79, M13.10, M13.111, M13.112, M13.119, M13.121, M13.122, M13.129, M13.131, M13.132, M13.139, M13.141, M13.142, M13.149, M13.151, M13.152, M13.159, M13.161, M13.162, M13.169, M13.171, M13.172, M13.179, M25.40, M25.411, M25.412, M25.419, M25.421, M25.422, M25.429, M25.431, M25.432, M25.439, M25.441, M25.442, M25.449, M25.451, M25.452, M25.459, M25.461, M25.462, M25.469, M25.471, M25.472, M25.473, M25.474, M25.475, M25.476, M25.48
Tickborne diseases transmitted by the same vector	B60.0, A77.40, A77.41, A77.49

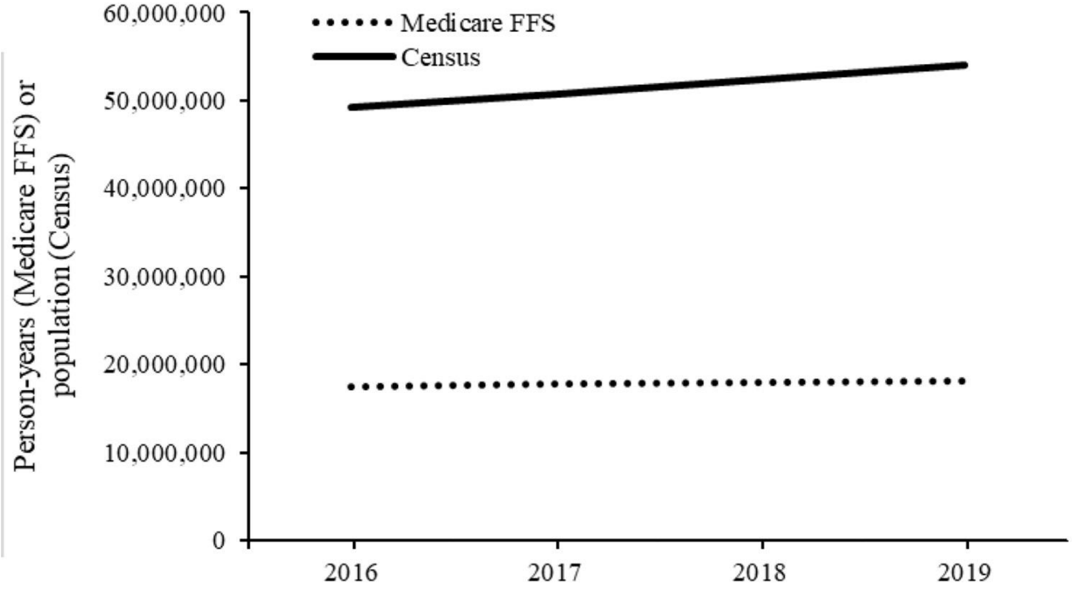

Appendix Figure 1. Total number of person-years in the Medicare fee-for-service (FFS) beneficiary population during the study period, as compared with the US Census population aged ≥65 years.

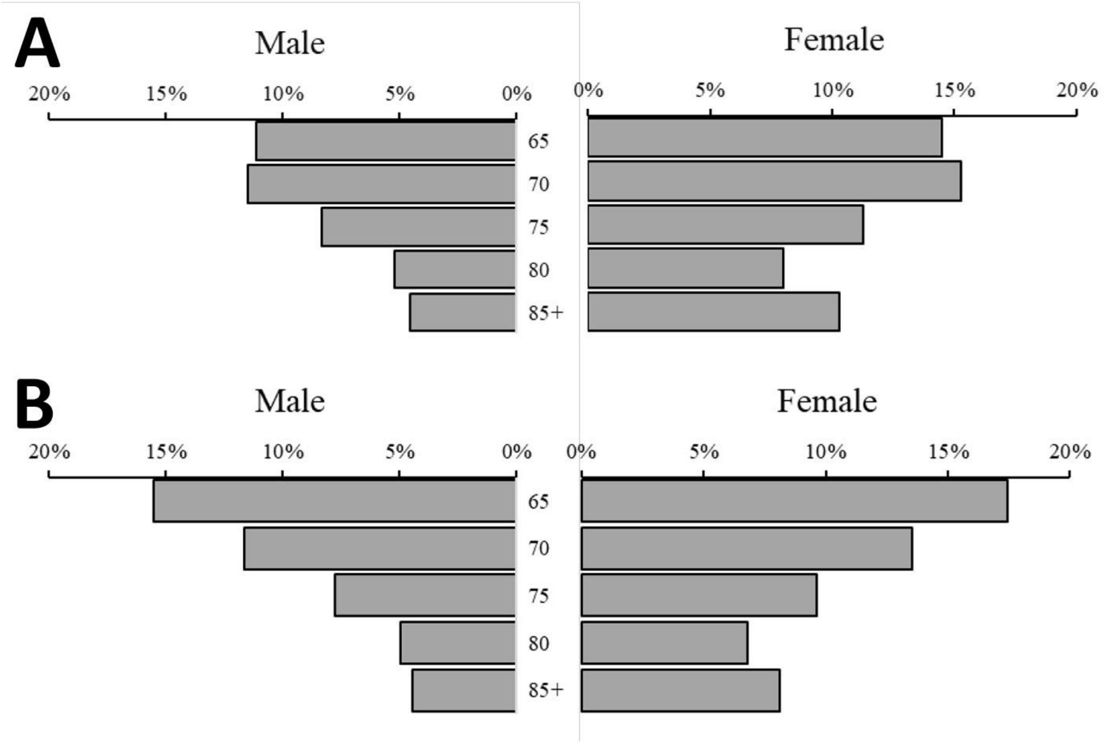

Appendix Figure 2. Percentage of Medicare fee-for-service (FFS) beneficiaries (A) and US Census population (B) by age group and sex, 2016–2019.

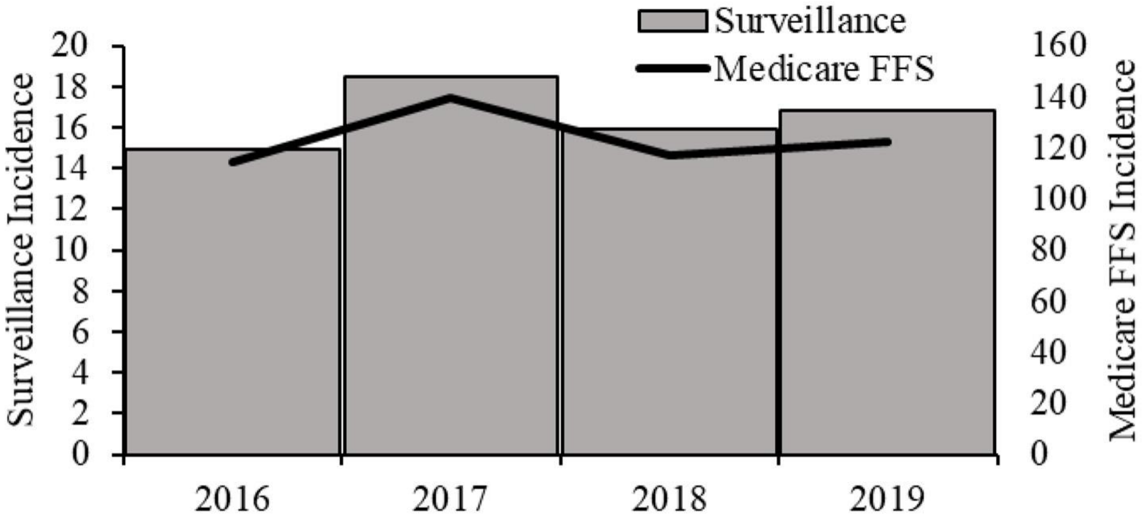

Appendix Figure 3. Incidence of Lyme disease diagnoses in Medicare fee-for-service (FFS) versus cases reported through surveillance, United States, 2016–2019. Incidence calculated as diagnoses per 100,000 beneficiaries in Medicare FFS or cases per 100,000 population among each surveillance subcategory.

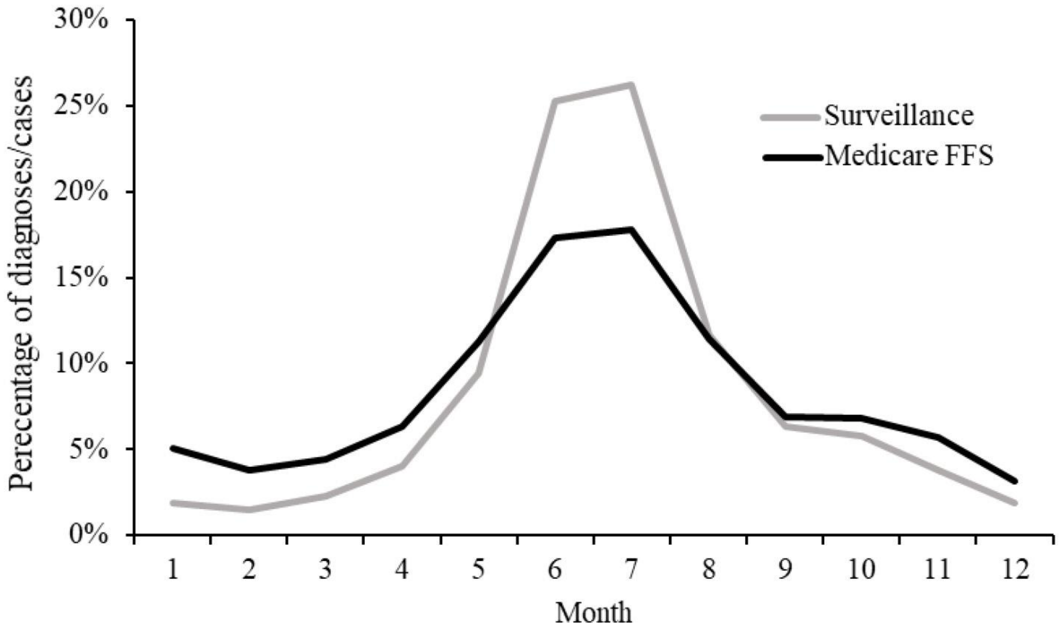

Appendix Figure 4. Percentage of Lyme disease diagnoses/cases by month in a study of the epidemiology of Lyme disease diagnoses among older adults, United States, 2016–2019.
